# Concurrent arterial and venous thrombosis in a patient with catastrophic antiphospholipid syndrome

**DOI:** 10.22088/cjim.12.0.487

**Published:** 2021

**Authors:** Muhammad Shoaib Khan, Muhammad Ishaq, Marek Siorek, Robert Biederman

**Affiliations:** 1 Department of Internal Medicine, Marshfield Clinic Health System, Marshfield, Wisconsin, USA; 2 Department of Interventional Radiology, Marshfield Clinic Health System, Marshfield, Wisconsin, USA; 3 Department of Cardiac MRI, Allegheny General Hospital, Pittsburgh, USA

**Keywords:** Antiphospholipid syndrome, Catastrophic antiphospholipid antibody syndrome, Thrombotic microangiopathies, Heparin induced thrombocytopenia, Disseminated intravascular coagulation

## Abstract

**Background:**

Antiphospholipid syndrome (APS) is marked by arterial, venous, or small vessel thrombosis. There have been few reported cases on APS presenting as thrombosis simultaneously involving large arteries and venous side of the blood circulation. CAPS can easily be confused with DIC, HIT, and other TMA. Anticoagulants remain the mainstay of treatment for CAPS, whereas in DIC and TMA, anticoagulants have no role.

**Case Presentation:**

A 43-year-old male presented to our facility with a chief complaint of right foot pain, calf cramps, and shortness of breath. The patient’s right dorsal pedal artery was not palpable. CT angiogram showed bilateral pulmonary emboli (fig.1), emboli within the right saphenofemoral artery (SFA), and popliteal artery (PA). Digital subtraction angiogram showed occlusive thrombi in SFA and in the PA. Thrombolysis was performed by an intra-arterial catheter-directed tissue plasminogen activator. Agitated saline bubble study showed no evidence of atrial shunting. The patient was noted to have thrombocytopenia, hypofibrinogenemia, high serum D-Dimer and normal activated partial thromboplastin time (APTT). The patient tested positive for anticardiolipin (aCL) antibodies and lupus anticoagulant (LA). After 12 weeks, aCL antibodies and LA testing were suggestive of APS.

**Conclusion:**

Simultaneous thrombosis in large arteries and veins is a very unusual presentation for the APS. The patients should be started on anticoagulants immediately as the mortality rate associated with CAPS is high and the key to management is initiating anticoagulants expeditiously.

Antiphospholipid syndrome (APS) is an autoimmune disorder typified by arterial, venous, or small vessel thrombosis (1). There have been few reported cases on APS presenting as thrombosis simultaneously involving both arterial and venous side of the blood circulation. Indeed, clinically, APS is thought to be predominantly a venous disorder quite infrequently affecting the arterial system. Catastrophic antiphospholipid syndrome (CAPS) is mostly the initial presentation of APS in as many as half of patients (2). CAPS can easily be confused with disseminated intravascular coagulation (DIC), heparin induced thrombocytopenia (HIT) and other thrombotic microangiopathies (TMA), therefore, recognizing the fine distinctive features between these disorders based on a thorough history, clinical presentation and laboratory results are important as their management is distinct. Anticoagulants remain the mainstay of treatment for CAPS (and APS).

## Case presentation

**Fig.1:**
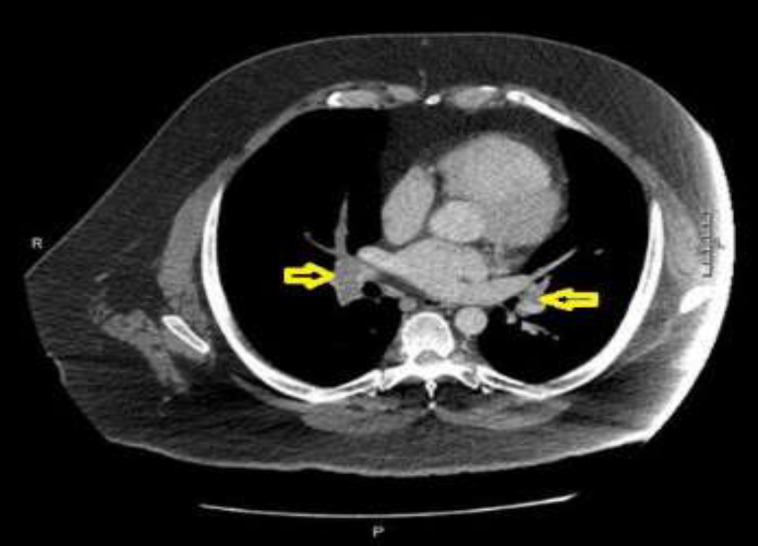
CT angiogram, axial view: Yellow arrows pointing at emboli (grey area) within pulmonary arteries

A 43-year-old male with a chief complaint of right foot pain, calf cramps, and shortness of breath presented to our facility. These sets of signs and symptoms started 3 days prior to the admission. On physical examination, patient’s right dorsalis pedal artery was not palpable and he appeared to have livedo reticularis like rash on upper and lower extremities. Vital signs indicated tachycardia, hypertension (171/93 mmHg at the time of presentation), tachypnea and the patient was hypoxic. CT angiogram showed extensive bilateral pulmonary emboli (fig.1), segmental occlusion of mid to distal right superficial femoral artery (SFA) and small emboli within right popliteal artery (PA). 

**Fig.2:**
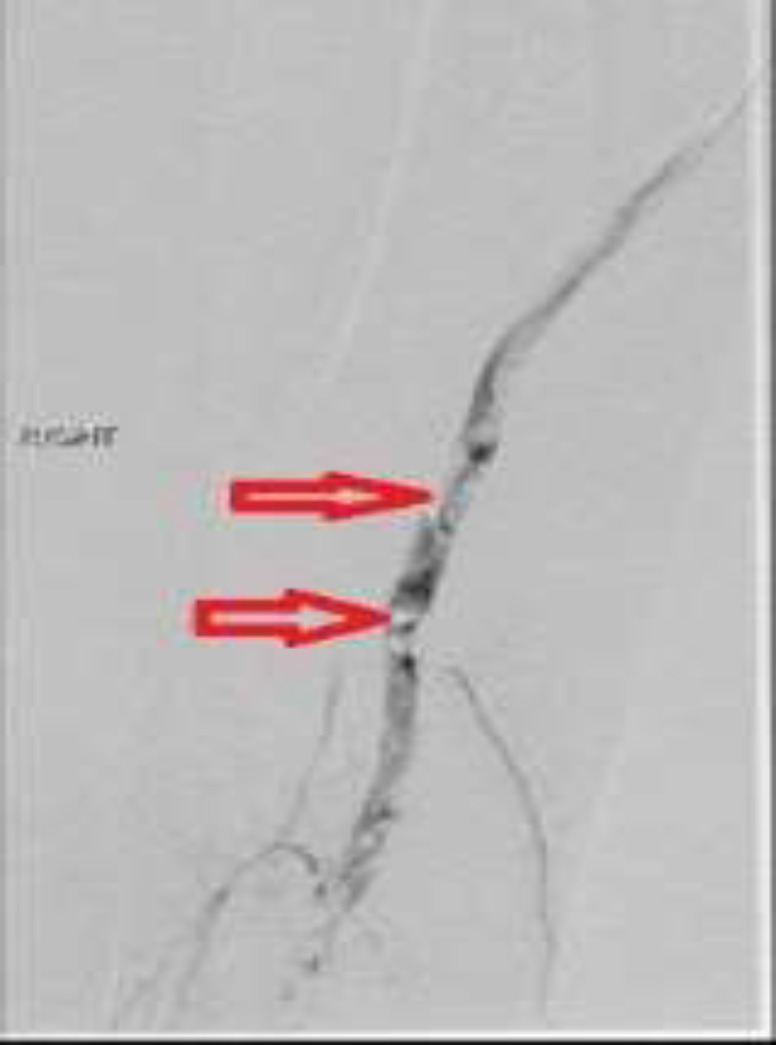
Digital subtraction angiogram of the distal superficial femoral artery and popliteal artery. Red arrows point at areas of filling defects (contrast appears dark)

The patient was initiated on a heparin drip. Digital subtraction angiogram performed later showed occlusive thrombi in the right SFA and the right PA (fig.2). Thrombolysis was performed by intra-arterial catheter directed TPA (tissue plasminogen activator) administration into the right common femoral artery. Following the procedure, the patient had palpable dorsalis pedal artery pulse. The agitated saline bubble study was unremarkable for any evidence of atrial shunting. The patient was noted to have a low platelet count of 102 x 10^3^/uL. Disseminated intravascular coagulation (DIC) panel revealed low serum fibrinogen (54 mg/dL), high serum D-Dimer and normal activated partial thromboplastin time (APTT). Because of suspicion of CAPS, we ordered anticardiolipin (aCL) antibodies and lupus anticoagulant (LA) testing, which came out to be suggestive of APS. aCL IgG and aCL IgM values came out to be 30.6 (GPL units) and 59.8 (MPL units), respectively. DRVVT came out to be 41.3 seconds (normal range= 29.2-37.8 seconds) and PTT came out to be 89.3 seconds (normal range= 51.9 to 78 seconds). Further inquiry from the patient revealed that his mother had clots in her blood vessels of the legs in her fourth decade of life, unfortunately, the patient did not know what her mother had been diagnosed with. For empiric management of probable CAPS, the patient was already on a heparin drip and was also initiated on 1 gram of IV methylprednisolone daily for three days. Heparin was bridged with warfarin before sending the patient home on warfarin. Coagulopathy workup performed by the primary care provider (PCP) was unremarkable for any evidence of anti-thrombin factor III, protein C, protein S, factor V Leiden and prothrombin gene abnormalities as causes of hypercoagulable state. After about 12 weeks, aCL antibodies and LA testing repeated by the PCP came out to be suggestive of APS. aCL IgG and IgM values came out to be 36.9 (GPL units) and 43.8 (MPL units), respectively.

## Discussion

APS is an autoimmune disorder marked by arterial, venous, or small vessel thrombosis and pregnancy related complications in the presence of antiphospholipid antibodies (1). APS is ‘Primary’ when there is no associated disorder, ‘Secondary’ when there is an associated autoimmune disorder such as systemic lupus erythematosus (SLE), and ‘Catastrophic’ when disseminated thrombosis occurs (3). According to Cervera et al., the most common presentations in APS patients are deep vein thrombosis (32%), thrombocytopenia (22%), livedo reticularis (20%), stroke (13%), superficial thrombophlebitis (9%), pulmonary embolism (9%), fetal loss (8%), and transient ischemic attack (7%) (4). We did extensive literature research and to our best knowledge, there have been few reported cases on APS presenting as thrombosis simultaneously involving both arterial and venous side of the blood circulation, especially the larger arteries of the body. Also, our patient presented with thrombosis of lower extremity arterial system, whereas the most common site of arterial thrombosis in APS is in the cerebral circulation (4). 

Our patient had thrombosis involving both arterial side (i.e SFA and PA) and venous side (i.e pulmonary arteries, which carry deoxygenated blood to the lungs and therefore represent like veins) of the circulation. The agitated saline echocardiogram using contrast injection for detecting right-to-left shunting via a patent foramen ovale (PFO) has a recognized association with thromboembolic events in younger patients (5). 

In our patient, there was no evidence for right to left shunting and, therefore, ruled out the possibility that the venous thrombi might have travelled from the venous system to the arterial system through atrial septum via a PFO. This raised the suspicion of hereditary coagulation abnormalities such as a protein C and S deficiency, factor V Leiden mutation or APS, however, all test results were unremarkable except for APS. CAPS is usually the initial manifestation of APS in nearly half of patients, while the remaining half have a history of APS (2). 

This seemed to be true for our patient as well who did not have any significant prior medical history. According to CAPS criteria (6) (table 1), our patient most likely had ‘probable CAPS’ as he had involvement of 4 organs/systems/tissues; lungs (respiratory distress associated with pulmonary embolism), arterial thrombosis (SFA and PFA involvement), venous thrombosis (pulmonary arteries), skin (livedo reticularis); simultaneous appearance of clinical manifestations in less than a week and laboratory confirmed APS antibodies (anticardiolipin antibodies and lupus anticoagulant). However, we did not do histopathological identification of small vessel occlusion in any one organ. By criteria, fulfilling 3 out of 4 criteria accounts to ‘probable CAPS (6). 

In addition to CAPS, other differential diagnoses in our patient were DIC, TMA and HIT. DIC is more likely to be associated with bleeding, and in acute DIC the prothrombin time (PT) and APTT are prolonged, whereas in our patient APTT was normal. TMA usually do not cause thromboses of large vessels, whereas our patient had thrombosis in large vessels. Lastly, HIT almost always occurs after exposure to heparin and is not associated with antiphospholipid antibodies.

**Catastrophic Antiphospholipid Antibody Syndrome Criteria**

- Features

A- Involvement of three or more organs, systems, and/or tissues

B- Simultaneous appearance of clinical features in less than one week

C- Establishing histopathological diagnosis of small vessel occlusion in at least one organ

D- Identification of Antiphospholipid antibodies 

- Classification

• Definite catastrophic APS

Presence of all four abovementioned features

• Probable catastrophic APS

All four criteria but only two organs, systems, and/or tissues are involved or

All four criteria but the laboratory identification of antibodies are not at least six weeks apart or

Criteria A, B, and D above or

A, C, and D and happening of a third event in more than a week but less than a month, despite being on anticoagulation

Recognizing these subtle differences in clinical presentation and laboratory results of these aforementioned disorders are important as their management is vastly different. Failure to recognize the subtleties will obligatorily have the potential for adverse clinical course. Anticoagulants remain the mainstay of treatment for CAPS (and HIT), whereas, in DIC and TMA there is limited or no role (respectively) of anticoagulants. Since the survival rate of catastrophic APS is approximately 50% (7), therefore, it is important to initiate anticoagulants expeditiously.

In conclusion simultaneous thrombosis in large arteries and veins is very unusual presentation for APS. Patients presenting with concomitant arterial and venous thrombosis require immediate work-up for hereditary thrombophilia such as APS, protein C and S deficiency. 

Once HIT and TMA are ruled, patients should be initiated on anticoagulants immediately as the mortality rate associated with CAPS is high and the key to management is the immediate recognition and institution of appropriate therapeutics. 
